# Treatment with Pterostilbene Ameliorates the Antioxidant Status of Bovine Spermatozoa and Modulates Cell Death Pathways

**DOI:** 10.3390/antiox13121437

**Published:** 2024-11-22

**Authors:** Christos Chavas, Vasiliki G. Sapanidou, Konstantinos Feidantsis, Sophia N. Lavrentiadou, Despoina Mavrogianni, Ioanna Zarogoulidou, Dimitrios J. Fletouris, Maria P. Tsantarliotou

**Affiliations:** 1 Laboratory of Animal Physiology, School of Veterinary Medicine, Faculty of Health Sciences, Aristotle University of Thessaloniki, GR-54124 Thessaloniki, Greece; cnchavas@vet.auth.gr (C.C.); vsapanid@vet.auth.gr (V.G.S.); slavrent@vet.auth.gr (S.N.L.); izarogo@vet.auth.gr (I.Z.); 2 Department of Fisheries & Aquaculture, School of Agricultural Sciences, University of Patras, GR-26504 Mesolonghi, Greece; 3 First Department of Obstetrics and Gynecology, Alexandra Hospital, Medical School, National and Kapodistrian University of Athens, GR-11528 Athens, Greece; dmavrogianni@med.uoa.gr; 4 Department of Hygiene and Technology of Animal Origin Products, School of Veterinary Medicine, Faculty of Health Sciences, Aristotle University of Thessaloniki, GR-54124 Thessaloniki, Greece; djflet@vet.auth.gr

**Keywords:** antioxidant, apoptosis, autophagy, mitochondria, oxidative stress, pterostilbene

## Abstract

Reactive Oxygen Species (ROS) play an important role in sperm physiology. They are required in processes such as capacitation and fertilization. However, the exposure of spermatozoa to ROS generated from internal or external sources may create a potentially detrimental redox imbalance. Antioxidant supplementation in semen is now a rather common approach to protect spermatozoa from oxidative stress (OS) during their handling and/or cryopreservation. Supplementation with pterostilbene, a potent antioxidant, protects spermatozoa from OS and ameliorates their post-thawing characteristics and viability. In the present study, we used freezing/thawing as a model of natural ROS overproduction and investigated the molecular mechanisms modulated by pterostilbene. Specifically, bovine frozen/thawed spermatozoa were incubated with 10 or 25 μM pterostilbene for 60 min. Results have shown that in a dose-independent manner, pterostilbene decreased lipid peroxidation and increased intracellular GSH levels. Moreover, pterostilbene ameliorated energy production, as ATP and AMP/ATP levels were restored, and increased autophagy levels through AMP-activated protein kinase (AMPK) activation, which finally resulted in the inhibition of apoptotic cell death in bovine spermatozoa when exposed to OS. This study sheds light on spermatozoa redox state, the crosstalk between apoptotic and autophagic pathways, and its role in determining the beneficial or detrimental effect of ROS in spermatozoa.

## 1. Introduction

Reactive oxygen species (ROS) are key players in sperm functions, such as maturation, capacitation, acrosome reaction, hyperactivation, and fertilization [1,2]. On the other hand, ROS are mediators of cell damage and death [3,4]. Therefore, it is important for spermatozoa to maintain a balance in the generation and scavenging of ROS in order to succeed in their role, which is the fertilization of the oocyte. In several cases of impaired fertility, supplementation of antioxidants either in vivo or in vitro has been implemented [5,6,7,8,9].

Apoptosis and autophagy are two highly conserved and closely intertwined processes that are modulated by ROS. Autophagy is induced relatively early in the stress process and if the cell has sufficient capacity to prevent the accumulation of damaged proteins and organelles (by recycling through lysosomal and proteasomal degradation), apoptosis is avoided [10]. The above is manifested in (a) the activation via phosphorylation of AMP-activated protein kinase (AMPK), an activator of autophagy, which is triggered by an increased AMP/ATP ratio [11], and (b) protein ubiquitination [12]. The latter is associated with the conversion of LC3 I to LC3 II) [3,13] and SQSTM1/p62 degradation, which lead to the formation of autophagosomes [14]. However, if the accumulated damage is beyond the cell’s ability for repair, the intrinsic bcl-2-regulated apoptotic pathway is activated [15], where the pro-apoptotic Bax [16] competes with the anti-apoptotic Bcl-2 [17]. The Bax/Bcl-2 ratio finally dictates the activation of caspases, thus activating apoptosis [18]. Although apoptosis has been studied extensively in spermatozoa, there are limited data on autophagy. The expression and function of autophagy-related proteins were demonstrated for the first time in human spermatozoa [19,20] and later in stallions [21]. Autophagy is activated by oxidative stress in ejaculated spermatozoa [22], while a cooperation between autophagy and apoptosis has been identified as a key factor in spermatogenesis [23,24]. However, to our knowledge, there are no reports regarding the role of autophagy in bovine spermatozoa or its implication in oxidative stress.

The process of freezing and thawing induces an overproduction of ROS in spermatozoa. Increased ROS levels in combination with low levels of intracellular antioxidants render spermatozoa susceptible to oxidative stress (OS) [25,26]. Moreover, seminal plasma is the main source of antioxidants, which spermatozoa are deprived of during certain assisted reproductive techniques (ARTs). Consequently, spermatozoa are exposed to higher levels of ROS compared to the levels found in the genital tract [27] and thus are at higher risk of suffering OS, which mediates the deterioration of their quality in general [28,29]. This is the result of alterations such as DNA fragmentation, lipid peroxidation, protein carbonylation, and mitochondria disruption, which largely deteriorate spermatozoa characteristics and may culminate in the apoptotic death of spermatozoa [30,31].

Pterostilbene (trans-3,5-dimethoxy-4-hydroxystilbene) is a non-flavonoid compound with anti-inflammatory, anti-apoptotic, and antioxidant properties [32]. Its antioxidant activity is mainly exerted through the scavenging of hydrogen peroxide (H_2_O_2_) and superoxide anions (O_2_^−^) [33] and the induction of the antioxidant defenses of cells, such as catalase, reduced glutathione, glutathione peroxidase, glutathione reductase, and superoxide dismutase [34,35,36]. The beneficial antioxidant effect of pterostilbene has been verified in various cell types [37,38,39] and bovine embryos [40]. However, the effect of pterostilbene on spermatozoa has only recently been investigated. We have previously demonstrated that the addition of pterostilbene into the medium during the in vitro handling of frozen/thawed spermatozoa prevents OS and preserves their quality parameters [8]. Specifically, two concentrations of pterosilbene were tested (10 and 25 μM). The concentration of 25 μM preserved motility and viability while reducing intracellular superoxide anion concentration. Moreover, pterostilbene facilitated acrosomal reactions in spermatozoa under capacitating conditions [8], but the underlying molecular mechanisms remain to be investigated. In this context, the present study employed frozen/thawed bovine spermatozoa as a model system for OS in order to elucidate the mechanisms that underlie the beneficial antioxidant effect of pterostilbene. Moreover, the results of this study can contribute to the improvement of semen preservation protocols, as these require an in-depth knowledge of gamete physiology and the biochemical processes occurring during semen collection, processing, and freezing/thawing [41].

## 2. Materials and Methods

### 2.1. Semen Samples

Semen from four (two Limousin, one Holstein, one Brown Swiss) healthy and sexually mature bulls of proven fertility with average age of 5 years was collected with the use of an artificial vagina. Two ejaculates were collected over a period of 10 min. The mean semen volume was 4.3 mL, with the minimum and maximum varying from 2.0 to 7.0 mL. The animals were housed at the Center of Artificial Insemination of Thessaloniki, Greece (License No. EL54SB01), National Ministry of Rural Development and Food. The samples were collected between November 2022 and January 2023. Only semen with >85% motility, <4% abnormalities, >70% viability, and >4 × 10^9^ spermatozoa mL^−1^ was used in this study. Each ejaculate was diluted with a home-made Tris-egg yolk extender (20% Tris-egg yolk, 7% glycerol, 78 mM citric acid, 69 mM fructose, 50 μg mL^−1^ tylosin, 250 μg mL^−1^ gentamycin, 150 μg mL^−1^ lincomycin, and 300 μg ml^−1^ spectinomycin) and packed into 0.5 mL plastic straws at a concentration of 50 × 10^6^ spermatozoa mL^−1^. The straws were then cooled to 4 °C for 4 h and were immediately transferred into a freezing chamber (Digital cool αlpha, IMV Technologies, Shanghai, China), where the temperature had been set to −12 °C. Subsequently, the straws were placed over a horizontal rack 3 cm above the surface of liquid nitrogen to reach −140 °C within 5 min. Finally, the straws were plunged into liquid nitrogen (−196 °C) for 5 min and transferred to sperm tanks (Figure S1). All straws were kept for 1 month before experimentation.

For each experiment, the appropriate number of straws were thawed via immersion in a water bath (37 °C, 40 s) and combined into a sterile conical tube (CellstarTubes, Greiner Bio One, Frickenhausen, Germany) to form a sperm pool. Spermatozoa were washed two times with Sperm Tyrode’s Albumin Lactate Pyruvate (TALP) solution (100 mM NaCl, 3.1 mM KCl, 25 mM NaHCO_3_, 0.29 mM NaH_2_PO_4_, 21.6 mM sodium lactate, 2 mM CaCl_2_, 1.5 mM MgCl_2_, and 10 mM HEPES sodium salt, supplemented with 1 mM sodium pyruvate and 50 μg mL^−1^ gentamycin) and centrifuged at 300× *g* for 10 min (25 °C). After each centrifugation, the supernatant was carefully removed, and the sperm pellet was resuspended in 1 mL Sperm TALP to repeat the process. The viability of sperm after centrifugation process was 55%. The concentration of spermatozoa in the final suspension was determined using a hemocytometer (OptikLabor, Grale HDS, Edgecliff, New South Wales, Australia). Spermatozoa were divided into three tubes. One tube served as a control, while the others were supplemented with two different concentrations (10 μM or 25 μM) of pterostilbene and incubated for 60 min at 37 °C. The stock solution of pterostilbene (20 mM) was prepared in dimethylosulfoxide (DMSO). The working solution of pterostilbene (500 μM) was freshly prepared before each experiment in Sperm TALP. An equal volume of medium was removed from the control group and was replaced by DMSO (vehicle) to a final concentration of 0.02%, which corresponds to the highest concentration of DMSO used in the treated groups. The experiment and all assays were repeated 6 times (n = 6).

### 2.2. Measurement of Lipid Peroxidation

Lipid peroxidation was evaluated on the basis of malondialdehyde (MDA) formation. MDA determination was carried out by a selective third-order derivative spectrophotometric method [42], slightly modified to suit spermatozoa analysis. In brief, 10^7^ spermatozoa, prepared as described above in a total volume of 50 μL, were supplemented with different concentrations of pterostilbene (0, 10, 25 μΜ) or DMSO. The samples were mixed with 50 μL of 5 mM FeSO_4_ (7H_2_O), diluted to a final volume of 3 mL with distilled water, and incubated for 60 min at 37 °C. After incubation, 500 μL trichloroacetic acid 35% (Panreac, Barcelona, Spain) and 2 mL butylated hydroxytoluene in hexane were added to the samples and were centrifuged for 1 min at 2000× *g*. The top hexane layer was discarded and 2.5 mL of the bottom aqueous layer was transferred to a new tube containing 1.5 mL of 0.8% aqueous 2-thiobarbituric acid. Following incubation at 70 °C for 30 min, the tubes were allowed to cool under tap water and submitted to third-order derivative spectrophotometry (Shimadzu UV 160A, Shimadzu, Kyoto, Japan). The height of the peak that appeared at 521.5 nm was used for the calculation of MDA concentration (ng/10^7^ spermatozoa) in the final extracts on the basis of slope and intercept data of the computed least squares fit of a freshly prepared calibration curve.

### 2.3. Determination of Total Antioxidant Capacity (TAC)

The total antioxidant capacity of spermatozoa was determined by a 2,2-Diphenyl-1-picrylhydrazyl radical (DPPH^•^) scavenging assay [43]. This method is based on the elimination of the stable free radical DPPH^•^. Antioxidants react with DPPH^•^, which is reduced to DPPH-H. By accepting hydrogen, the solution loses the characteristic deep purple color, and the discoloration (lower absorbance) is proportional to scavenging capacity of the compound. Spermatozoa (5 × 10^6^) were prepared as described above to remove the cryoprotectants and were supplemented with different concentrations of pterostilbene (0, 10, 25 μΜ) or DMSO. After 60 min of incubation (37 °C), the samples were centrifuged at 300× *g* for 10 min (25 °C) and resuspended in TAC Phosphate Buffer (10 mM KH_2_PO_4_, 10 mM Na_2_HPO_4_, pH 7.4). Spermatozoa were subjected to two cycles of sonication at 28 kHz for 60 s. Subsequently, DPPH^•^ (0.08 mM) was added and the samples were incubated at room temperature (RT), in the dark, for 60 min. The tubes were centrifuged for 5 min at 2000× *g* and the absorbance was measured at 517 nm using a spectrophotometer (Pharmacia LKB-Novaspec II, Northwich, Cheshire, UK). TAC was determined as the % of reduced DPPH^•^ relative to the control, which was set as 100%.

### 2.4. Determination of Intracellular Glutathione (GSH)

To determine the reduction in intracellular glutathione (GSH) levels, spermatozoa lysates (20 × 10^6^/reaction) were prepared as described for the determination of TAC. The lysates were incubated with 0.33 mM DTNB [5,5′-dithiobis (2-nitrobenzoic acid)] [44]. The thiol groups of GSH in the lysates cleave the disulfide bond in DTNB to yield 2-nitro-5-thiobenzoic acid, which ionizes to the TNB^2−^ dianion, which has a yellow color and can be quantified at 412 nm using a spectrophotometer (Pharmacia LKB-Novaspec II, Northwich, Cheshire, UK). The results were expressed as percentage (%) of the control, which was set as 100%.

### 2.5. Determination of ATP and AMP

The pool of thawed spermatozoa was layered onto discontinuous Percoll gradients (45% and 80%) and centrifuged (380× *g*, 25 min, RT) to remove the cryoprotectants. The supernatant was carefully removed, and the pellet was washed with Sperm TALP, as described above. For ATP and AMP determination, the protocols by Söderquist and Stålhammar [45] and Manfredi et al. [46] were applied with some modifications. In particular, frozen spermatozoa were homogenized using ice-cold 0.6 M perchloric acid (PCA), containing 150 mM EDTA and the homogenates were centrifuged (10,000× *g*, 4 °C, 10 min) [47,48]. Neutralized, deproteinized PCA extracts were stored at −80 °C and used to determine concentrations of metabolites using standard spectrophotometric NADH or NADPH-linked enzymatic assays at an absorbance wavelength of 340 nm [49,50]. Protein concentrations were determined using the BioRad protein assay (Bio-Rad Protein Assay Kit, 5000001, Hercules, CA, USA).

### 2.6. SDS-PAGE/Immunoblot and Dot Blot Analysis

Percoll gradient-isolated spermatozoa, as previously described, were homogenized in 50 μL cold lysis buffer (20 mM β-glycerophosphate, 50 mM NaF, 2 mM EDTA, 20 mM Hepes, 0.2 mM Na_3_VO_4_, 10 mM benzamidine, pH 7, 200 μM leupeptin, 10 μΜ trans-epoxy succinyl-L-leucylamido-(4-guanidino)butane, 5 mM dithiotheitol, 300 μΜ phenyl methyl sulfonyl fluoride (PMSF), 50 μg mL^−1^ pepstatin, and 1% *v*/*v* Triton X-100) and were centrifuged (10,000× *g*, 10 min, 4 °C) after they had been extracted on ice for 30 min. Protein concentrations were determined using the BioRad protein assay (Bio-Rad Protein Assay Kit, 5000001, Hercules, CA, USA). The supernatants were mixed 3/1 (*v*/*v*) with sample buffer (330 mM Tris-HCl, 13% *v*/*v* glycerol, 133 mM DTT, 10% *w*/*v* SDS, 0.2% *w*/*v* bromophenol blue) and completely heat-denatured at 100 °C.

Bax, Bcl-2, phospho-AMPK, AMPK, LC3 II/I, SQSTM1/p62, and β-actin levels were determined in the samples according to well-established protocols for SDS-PAGE/immunoblot analysis. Equivalent amounts of proteins (50 μg) were separated on 10% (*w*/*v*) acrylamide and 0.275% (*w*/*v*) bisacrylamide slab gels, and transferred electrophoretically onto nitrocellulose membranes (0.45 μm, Schleicher & Schuell, Keene, NH 03431, USA). Cleaved caspase and ubiquitin conjugate levels were determined by dot blot. The samples were diluted to a concentration of 5 μg mL^−1^ in 150 mM NaCl; 100 μL was loaded onto a pre-soaked nitrocellulose membrane (0.45 μm) in a dot blot vacuum apparatus (BioRad, Bio-Dot^®^ Microfiltration System, 1703938, Hercules, CA, USA) and gravity-fed through the membrane.

We employed 5% (*w*/*v*) non-fat milk in TBST (20 mM Tris-HCl, pH 7.5, 137 mM NaCl, 0.1% (*v*/*v*) Tween 20) for 30 min at RT to block non-specific binding sites on the membranes. Then, the nitrocellulose membranes were treated with the following antibodies in dilutions recommended by the manufacturer guidelines: anti-Bcl2 (2872, Cell Signaling, Beverly, MA, USA), anti-Bax (B-9) (2772, Cell Signaling, Beverly, MA, USA), anti-phospho AMPK (2535, Cell Signaling, Beverly, MA, USA), anti-AMPK (5831, Cell Signaling, Beverly, MA, USA), anti-p62/SQSTM1 (5114, Cell Signaling, Beverly, MA, USA), anti-LC3B (3868, Cell Signaling), anti-cleaved caspase antibody (8698 Cell Signaling, Beverly, MA, USA), and anti-ubiquitin antibody (Cat. No. 3936, Cell Signaling, Beverly, MA, USA). Actin (anti-β actin 3700, Cell Signaling, Beverly, MA, USA) was employed for quality transfer control and normalization.

After washing in TBST (3 periods, 5 min each), the blots and dots were incubated with the appropriate horseradish peroxidase-linked secondary antibody and washed again in TBST (3 periods, 5 min each time), and the bands were detected using enhanced chemiluminescence (Chemicon, Rolling Meadows, IL, USA) with exposure to Fuji Medical X-ray films. Films were quantified by laser-scanning densitometry (GelPro Analyzer Software, GraphPad, Version 3.0.00.00 https://www.graphpad.com).

### 2.7. Statistics

One-way analysis of variance (ANOVA) (GraphPad Instat 3.0) followed by Bonferroni post hoc were employed to test for significance at *p* < 0.05 (5%) level between all experimental groups (control, P10, P25) examined herein.

## 3. Results

### 3.1. Oxidative Stress/Antioxidant Status

**Figure 1:**
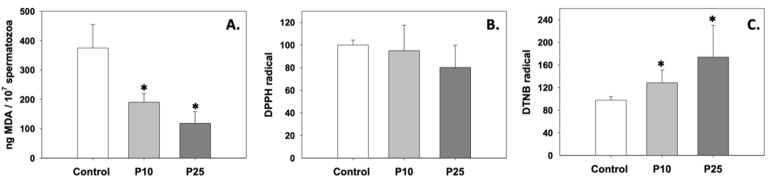
MDA (**A**), total antioxidant capacity (**B**), and intracellular GSH (**C**) levels in bovine spermatozoa under the effect of 10 μM (P10) or 25 μM (P25) pterostilbene treatments. Values constitute means ± S.D. Asterisks (*) denote statistically significant differences compared to control (*p* < 0.05, n = 6). No statistically significant differences were found between the P10 and P25 groups.

Oxidative stress in terms of lipid peroxidation was significantly inhibited, since MDA levels decreased compared to control (*p* < 0.05) under the effect of both P10 and P25 treatments (Figure 1A). While the P10 and P25 treatments had no effect on the total antioxidant capacity of spermatozoa (Figure 1B), both concentrations significantly (*p* < 0.05) increased intracellular GSH levels relative to the control group (Figure 1C). The effect of pterostilbene on the aforementioned parameters was dose-independent (*p* > 0.05).

### 3.2. Apoptosis

**Figure 2:**
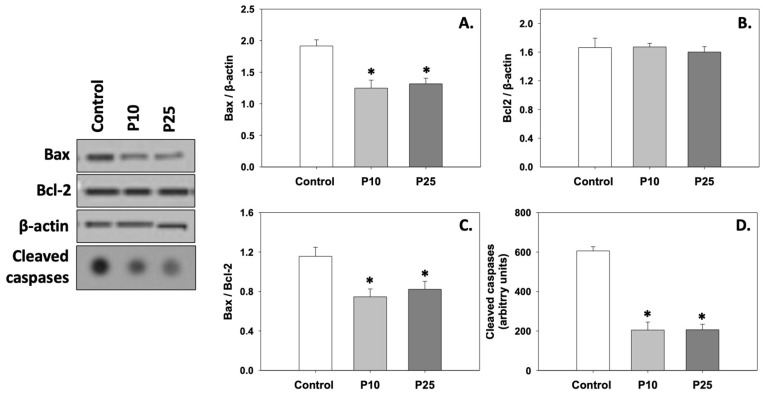
Bax (**A**), Bcl-2 (**B**), Bax/Bcl-2 (**C**), and cleaved caspase (**D**) levels in bovine spermatozoa in the presence of 10 μM (P10) or 25 μM (P25) pterostilbene. Values constitute means ± S.D. Spermatozoa extracts from control, P10, and P25 groups were immunoblotted for Bax, Bcl-2, and cleaved caspases. The levels of β-actin were determined to verify equal loading. Representative blots are shown (Figures S2 and S3). Asterisks (*) denote statistically significant differences compared to the control (*p* < 0.05, n = 6). No statistically significant differences were found between P10 and P25 groups.

Figure 2A depicts the levels of Bax, which were significantly (*p* < 0.05) decreased compared to the control under the effect of both P10 and P25 treatments. Both concentrations had a similar effect on Bax. On the other hand, Bcl-2 levels remained unchanged under both P10 and P25 treatments compared to the control (Figure 2B). Therefore, the Bax/Bcl-2 ratio (Figure 2C) was reduced, and cleaved caspase levels (Figure 2D) significantly decreased (*p* < 0.05) in both P10 and P25 treatment groups. No statistically significant differences were observed between the two concentrations of pterostilbene (P10 and P25). Judging from the above, both P10 and P25 treatments exhibit a similar anti-apoptotic effect.

### 3.3. Energy Content

**Figure 3:**
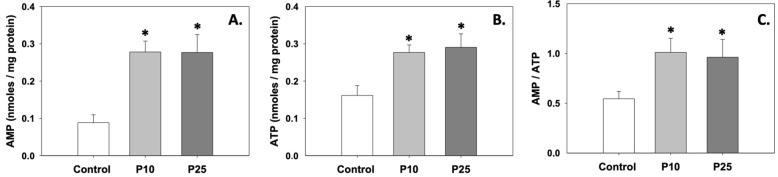
AMP (**A**) and ATP (**B**) levels and AMP/ATP (**C**) ratios in bovine spermatozoa treated with 10 μM (P10) or 25 μM (P25) pterostilbene. Values constitute means ± S.D. Asterisks (*) denote statistically significant differences compared to the control (*p* < 0.05, n = 6). No statistically significant differences were found between P10 and P25 groups.

Figure 3A,B depict the levels of AMP and ATP, which were significantly (*p* < 0.05) increased compared to the control under the effect of both P10 and P25 treatments. Likewise, the AMP/ATP ratio was also increased (*p* < 0.05) in the presence of pterostilbene compared to the control (Figure 3C). Regarding AMP and ATP levels and the AMP/ATP ratio, no statistically significant differences were observed between the two treatments.

### 3.4. AMPK Phosphorylation

**Figure 4:**
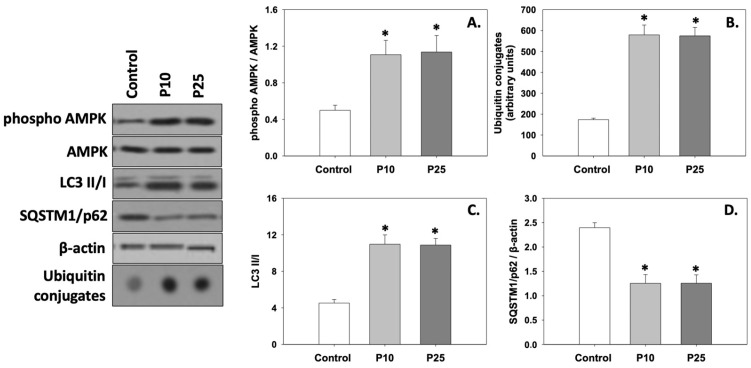
Phospho AMPK/AMPK ratio (**A**), ubiquitin conjugates (**B**), LC3 II/I ratio (**C**), and SQSTM1/p62 (**D**) levels in bovine spermatozoa exposed to 10 μM (P10) or 25 μM (P25) pterostilbene treatments. Values constitute means ± S.D. Spermatozoa extracts from control, P10, and P25 groups were immunoblotted for phospho AMPK, AMPK, ubiquitin conjugates, LC3, and SQSTM1/p62. The levels of β-actin were determined to verify equal loading. Representative blots are shown (Figures S4–S7). Asterisks (*) denote statistically significant differences compared to the control (*p* < 0.05, n = 6). No statistically significant differences were found between P10 and P25 groups.

Figure 4A depicts the levels of AMPK phosphorylation (phospho AMPK/AMPK ratio). Both concentrations of pterostilbene significantly (*p* < 0.05) increased the phosphorylation of AMPK compared to the control. No statistically significant differences were observed between the two treatments.

### 3.5. Autophagy

Both ubiquitin conjugates (Figure 4B) and the LC3 II/I ratio (Figure 4C) were significantly (*p* < 0.05) increased in the presence of either the P10 or P25 treatment compared to the control. On the other hand, both concentrations of pterostilbene significantly (*p* < 0.05) decreased SQSTM1/p62 (Figure 4D) levels compared to the control. No statistically significant differences were observed between the two treatments. Therefore, it seems that both concentrations of pterostilbene increase and ameliorate the levels of autophagy, which may be dysregulated in frozen/thawed spermatozoa (control).

## 4. Discussion

The data of the present study show that spermatozoa compensate for freeze/thaw-induced OS when they are treated with pterostilbene. In a previous study, we demonstrated that the addition of 25 μM pterostilbene in bovine sperm preparation media ameliorated the motility, viability, intracellular superoxide anion concentration, and acrosomal status of spermatozoa [8]. In the present study, we hypothesized that spermatozoa under OS, as in the case of freezing/thawing, sustain a disruption of the energy production mechanism, which may have an impact on the normal autophagic process and/or apoptosis. The present results showed that pterostilbene scavenged ROS and normalized the levels of energy production and turnover, as indicated by the increased ATP, GSH, and AMPK phosphorylation levels, as well as the increased AMP/ATP ratio. Subsequently, the changes in autophagic indicators examined herein evidence the increase in autophagy in pterostilbene-treated spermatozoa, which in turn suppresses apoptosis. The above hypothesis is also reflected by the mitigation of stress-induced motility and viability by pterostilbene [8]. Although a small number of animals were included in this study, the sufficient number of repeats and the statistically significant results obtained support the validity of the acquired data. Moreover, the protective effects of pterostilbene on post-thawed spermatozoa is similar to the effect of other molecules with antioxidant properties studied by our group, namely crocin [51], crocetin [52], erythropoietin [9], and melatonin [53].

To our knowledge, no studies regarding the effect of pterostilbene on the antioxidant response, energy production, and cell death pathways of bovine spermatozoa exist. This study provides, for the first time, an integrated picture of its advantageous effects on the cellular responses of stressed bovine spermatozoa. No differences between the two examined concentrations (10 μM and 25 μM) of pterostilbene were found in the present study, implying that the maximal effect of pterostilbene is already reached at the lower concentration of 10 μM. This enhancement is linked to the antioxidant activity of pterostilbene, which has been associated with cancer prevention, modulation of neurological disorders, anti-inflammatory effects, reduction in vascular disease, and improvement in diabetes [35].

Although the exact scavenging mechanism of pterostilbene is not known, we can hypothesize that its ameliorating activity on spermatozoa is initiated by the restoration of normal mitochondrial function [8]. Its antioxidant role probably shunts ROS overproduction in the mitochondrial inner membrane [8], which increases its potential during the cryopreservation and freezing/thawing of spermatozoa [53], resulting in an excess production of several ROS [54,55]. This overproduction of ROS impairs several processes and leads to DNA fragmentation and acrosomal damage [55]. The resulting OS is mostly manifested in spermatozoa by increased susceptibility to lipid peroxidation and the degradation of several cellular structures [56,57]. The results of the present study are in line with other studies where the freeze/thaw process seems to increase lipid peroxidation damage, which may be further enhanced due to the decrease in antioxidant defense [8,51,52,58]. The enzymatic antioxidant system, e.g., GSH, glutathione peroxidase, catalase, and superoxide dismutase, comprises the first line of defense against OS in order for spermatozoa to maintain their motility and viability [59,60]. However, this endogenous mechanism may be insufficient for the prevention of oxidative damage, especially during prolonged storage [61].

As previously mentioned, we assumed that pterostilbene’s ameliorating effect on spermatozoa starts from the mitochondrial restoration of the electron transport chain (ETC) [8]. Although mitochondria are a major source of ROS [62], changes in the structure of the mitochondrial inner membrane during oxidative conditions impair the functionality of the ETC, resulting in energy failure and ultimately leading to cell death [63,64]. Overall, the results of the present study provide evidence of ATP depletion in spermatozoa due to freeze/thaw-induced OS. Similarly, Cardoso et al. [65] found that upon OS induced by ascorbate and iron, the mitochondrial respiratory chain complexes were significantly affected and therefore ATP levels were markedly decreased. Goldstein et al. [66] suggested that •OH radicals damage these peptides in the presence of transition metal ions. Evolutionarily far from mammal cells, ATP depletion has also been observed in the Mediterranean mussel *Mytilus galloprovincialis* (Linnaeus, 1758) when oxidative damage was set due to thermal stress [67]. In the present study, the addition of pterostilbene to thawed spermatozoa seemed to re-establish normal ATP production and turnover, as also exhibited by the increased ATP, AMP, and AMP/ATP levels after treatment with pterostilbene. Similarly, the activity of the mitochondrial respiratory chain complexes and ATP production were restored in cortical synaptosomes sustaining OS when they were incubated in the presence of other antioxidants, namely idebenone, reduced glutathione (GSH), or a combination of vitamin E, idebenone, and GSH [65,68].

The insufficiency of endogenous enzymatic antioxidant mechanisms to adequately suppress OS effects triggers the onset of downstream signaling targets such as apoptosis [69,70]. Additionally, it is highly probable that frozen/thawed spermatozoa sustain OS and undergo apoptosis following the induction of both the Bax/Bcl-2 ratio and caspase cleavage [71]. It has also been demonstrated that repeated freezing/thawing increases pro-apoptotic Bax and decreases anti-apoptotic Bcl-2 levels in bovine spermatozoa [72]. A direct association between increased sperm damage caused by ROS and high levels of cytochrome C, and caspase 9 and 3 has been demonstrated [72,73]. The results of the present study are in line with our previous data, which showed increased apoptotic events in bovine spermatozoa before the application of erythropoietin, which ameliorated the antioxidant status of spermatozoa [9].

The results of the present study seem to support the anti-apoptotic role of autophagy [3,10,74,75], since they demonstrate that pterostilbene prevents the induction of apoptosis via an increase in the autophagic levels. The increase in autophagy in spermatozoa treated with pterostilbene is reflected in all the corresponding cellular events: increased AMPK phosphorylation, increased ubiquitination, increased LC3 conversion, and increased SQSTM1/p62 degradation. The beneficial role of another antioxidant, crocin, in *Rattus norvegicus* myocardium and isolated cardiomyocytes that were exposed to OS generated by STZ diabetes or high glucose, respectively, is reflected in the normalization of dysregulated—due to OS—autophagy and its anti-apoptotic role [76]. The dual role of autophagy as a cell death executioner and protector highlights the complexity of the interactions between the apoptotic and autophagic machinery. However, a strong causal relationship wherein one process controls the other has not been adequately demonstrated [77]. OS due to freezing/thawing may decrease autophagy levels in spermatozoa. Aparicio et al. [78] have demonstrated that the autophagy-related protein LC3 is modulated in stallion spermatozoa during freezing/thawing-induced OS. Finally, the above results identify pterostilbene as a good candidate for the improvement of cryopreservation protocols. Similar to crocin, which when added to the cryopreservation medium protects spermatozoa from freezing/thawing-induced oxidative stress [58], pterostilbene could also be tested in future studies for its potential role as a cryoprotectant antioxidant.

## 5. Conclusions

**Figure 5:**
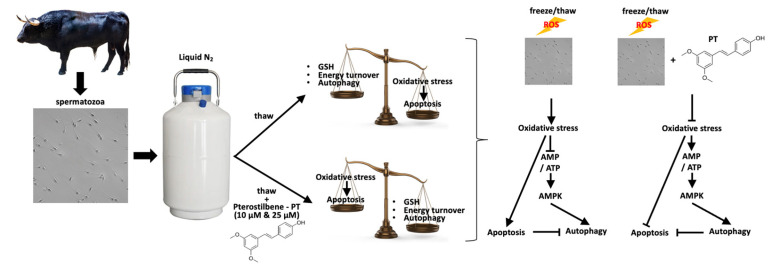
Summarized model of pterostilbene’s effect on biochemical and physiological stress responses in bovine spermatozoa exposed to oxidative stress.

The present study complements the existing results regarding the effect of pterostilbene on sperm quality parameters [8] and sheds light on the molecular mechanisms that mediate the protective effect of pterostilbene on bovine spermatozoa exposed to OS due to freezing/thawing. Pterostilbene, by enforcing the antioxidant status of spermatozoa, affects the balance between apoptosis and autophagy by enforcing the latter (Figure 5). Considering the advantageous properties of pterostilbene (simple extraction methods, high bioavailability, high antioxidant potency), the present findings are of great interest and set the ground for future studies focusing on the direct relationship between sperm capacitation and ROS-regulated autophagy.

## Data Availability

Data are contained within the article or Supplementary Materials.

## Supplementary Materials

The following supporting information can be downloaded at https://www.mdpi.com/article/10.3390/antiox13121437/s1, Figure S1: Freezing curve; Figure S2: The complete original immunoblots shown in Figure 2 regarding Bax, Bcl-2, and β-actin are presented in order below. The individual parts comprising Figure 2 are specified using black boxes; Figure S3: The complete original immunoblots shown in Figure 2 regarding cleaved caspases are presented in order below. The individual parts comprising Figure 2 are specified using black boxes; Figure S4: The complete original immunoblots shown in Figure 4 regarding phospho AMPK and AMPK are presented in order below. The individual parts comprising Figure 4 are specified using black boxes; Figure S5: The complete original immunoblots shown in Figure 4, regarding SQSTM1/p62 and LC3 II/I, are presented in order below. The individual parts comprising Figure 4 are specified using black boxes; Figure S6: The complete original immunoblots shown in Figure 4 regarding β-actin are presented in order below. The individual parts comprising Figure 4 are specified using black boxes; Figure S7: The complete original immunoblots shown in Figure 4 regarding ubiquitin conjugates are presented in order below. The individual parts comprising Figure 4 are specified using black boxes.
