# Supplementary material for: Treatment with Pterostilbene Ameliorates the Antioxidant Status of Bovine Spermatozoa and Modulates Cell Death Pathways
Source: Antioxidants (Basel). 2024 Nov 22;13(12):1437. doi: 10.3390/antiox13121437 (PMC11672693; doi:10.3390/antiox13121437)

Treatment with pterostilbene ameliorates the antioxidant status of bovine spermatozoa and modulates cell death pathways

Christos Chavas[1], Vasiliki G. Sapanidou[1], Konstantinos Feidantsis[2], Sophia N. Lavrentiadou[1], Despoina Mavrogianni[3], Ioanna Zarogoulidou[1], Dimitrios J. Fletouris[4] and Maria P. Tsantarliotou[1]

[1]Laboratory of Animal Physiology, School of Veterinary Medicine, Faculty of Health Sciences, Aristotle University of Thessaloniki, GR-54124 Thessaloniki, Greece

[2]Department of Fisheries & Aquaculture, School of Agricultural Sciences, University of Patras, GR-26504 Mesolonghi, Greece

[3]First Department of Obstetrics and Gynecology, Alexandra Hospital, Medical School, National and Kapodistrian University of Athens, GR-11528 Athens, Greece

[4]Department of Hygiene and Technology of Animal Origin Products, School of Veterinary Medicine, Faculty of Health Sciences, Aristotle University of Thessaloniki, GR-54124 Thessaloniki, Greece

Figure S1: freezing curve

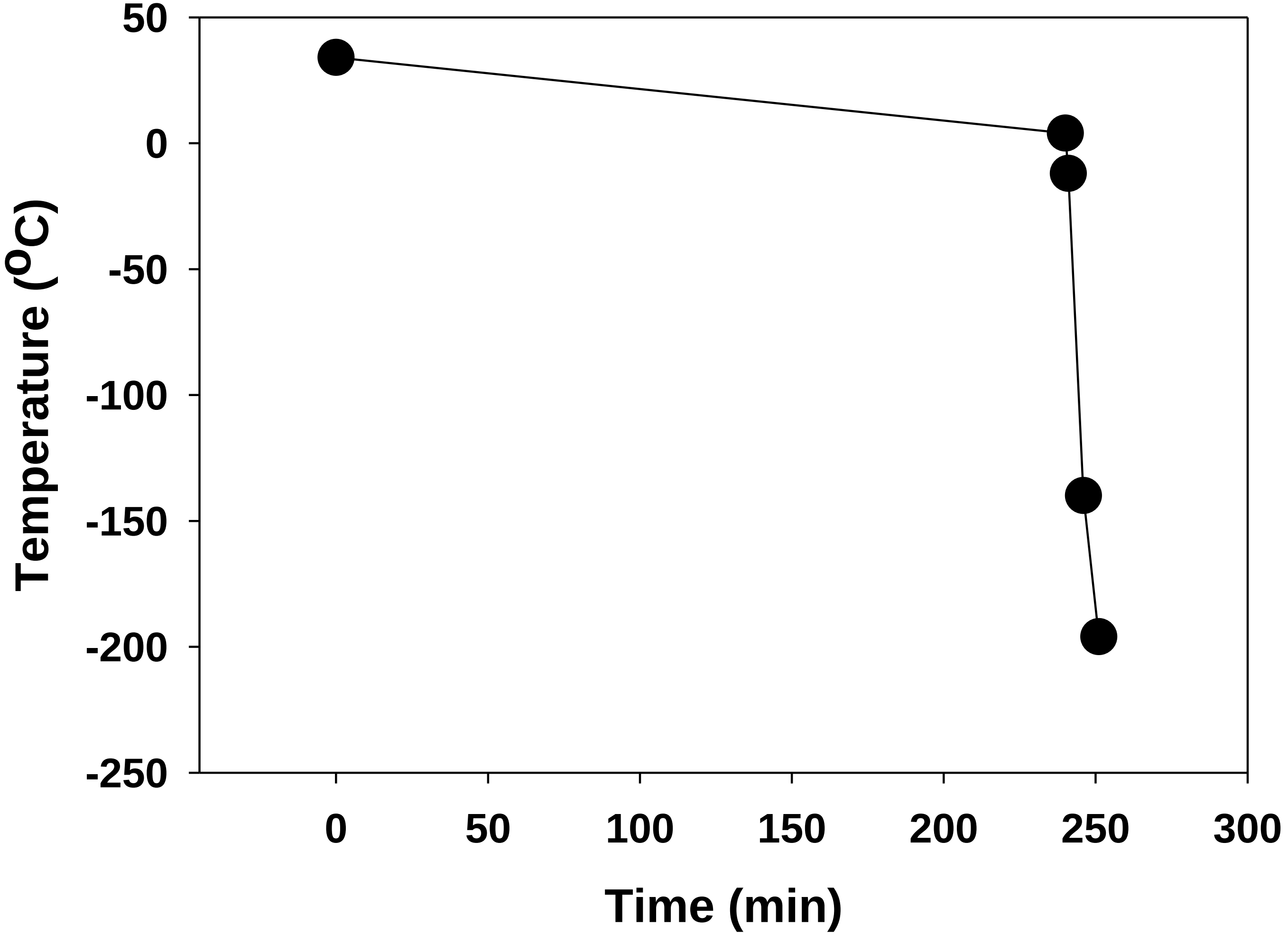

Figure S2: The complete original immunoblots shown in Figure 2 regarding Bax, Bcl-2 and β-actin are presented in order below. The individual parts comprising Figure 2 are specified using black boxes.

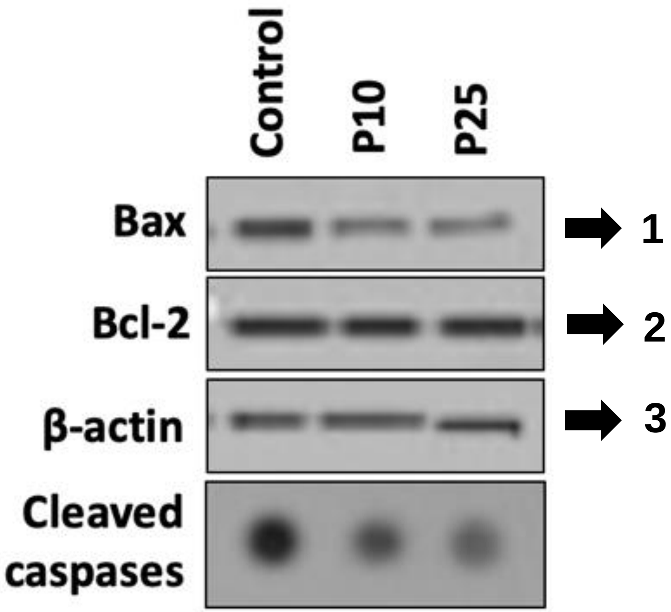

Full unedited immunoblot for figure 2

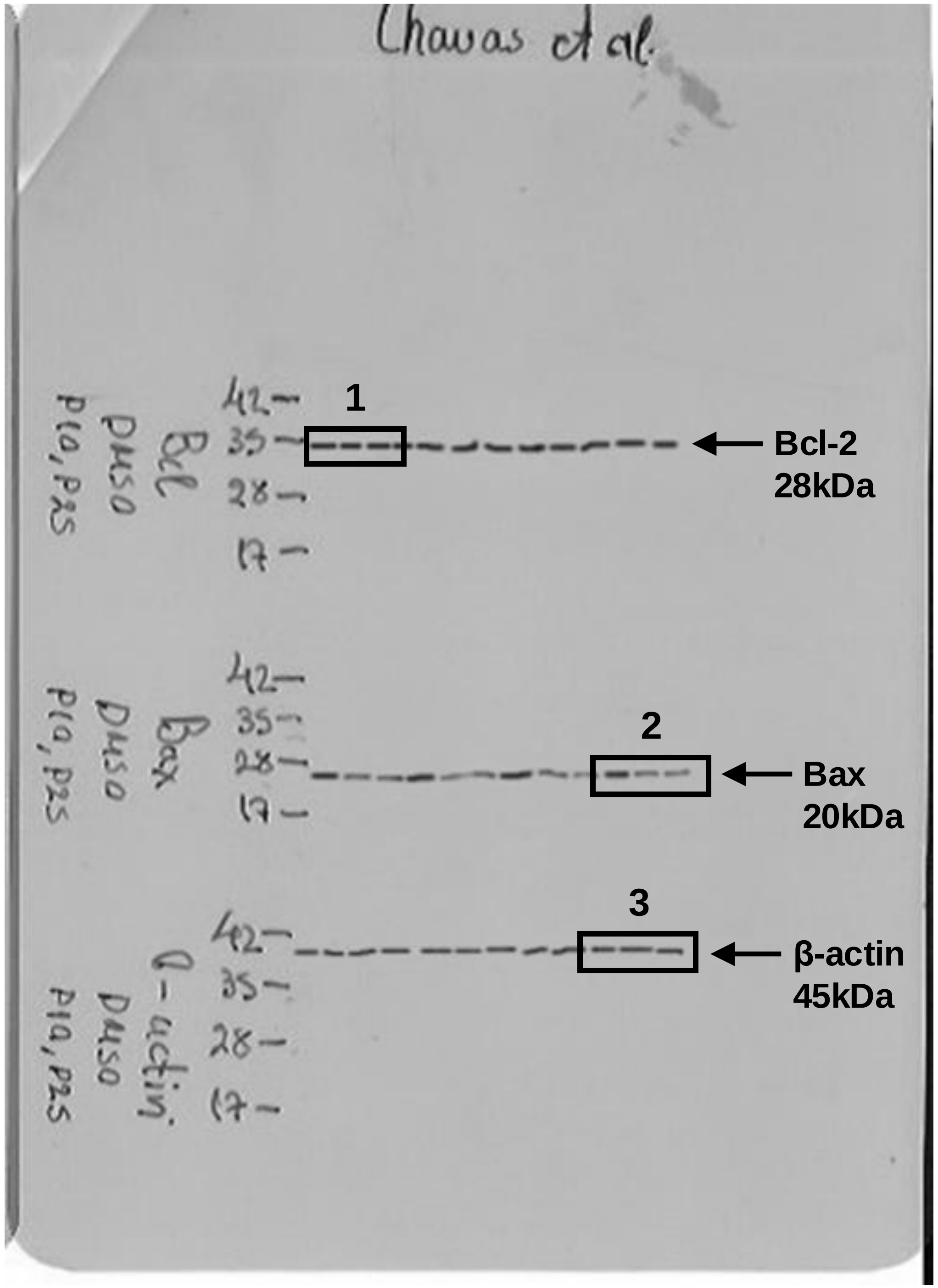

Figure S3: The complete original immunoblots shown in Figure 2 regarding cleaved caspases are presented in order below. The individual parts comprising Figure 2 are specified using black boxes.

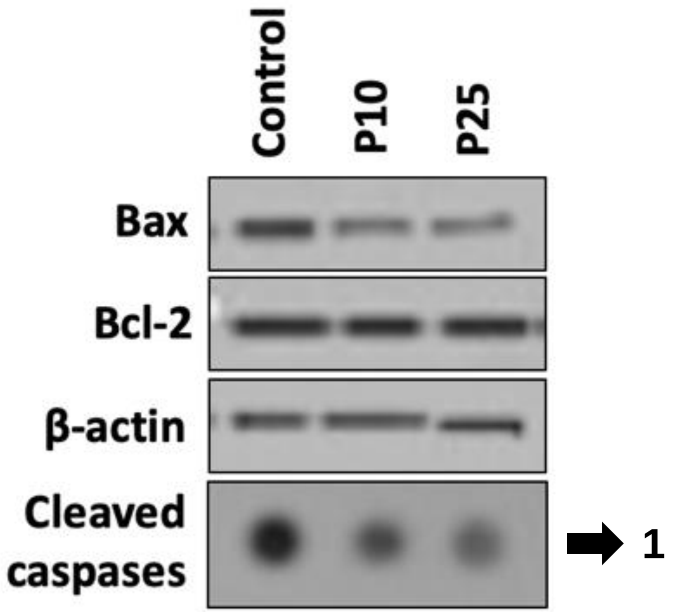

Full unedited immunoblot for figure 2

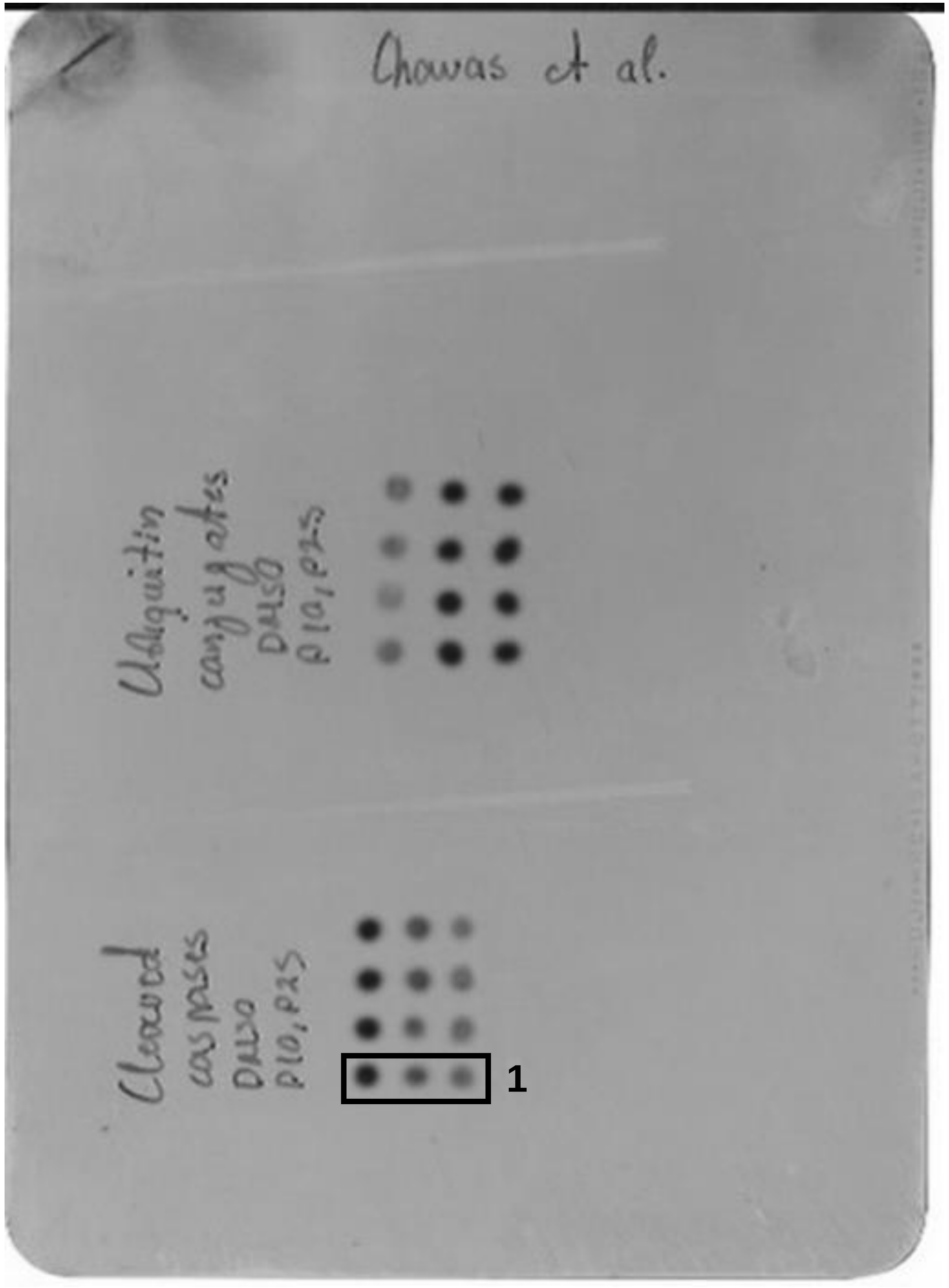

Figure S4: The complete original immunoblots shown in Figure 4 regarding phospho AMPK and AMPK are presented in order below. The individual parts comprising Figure 4 are specified using black boxes.

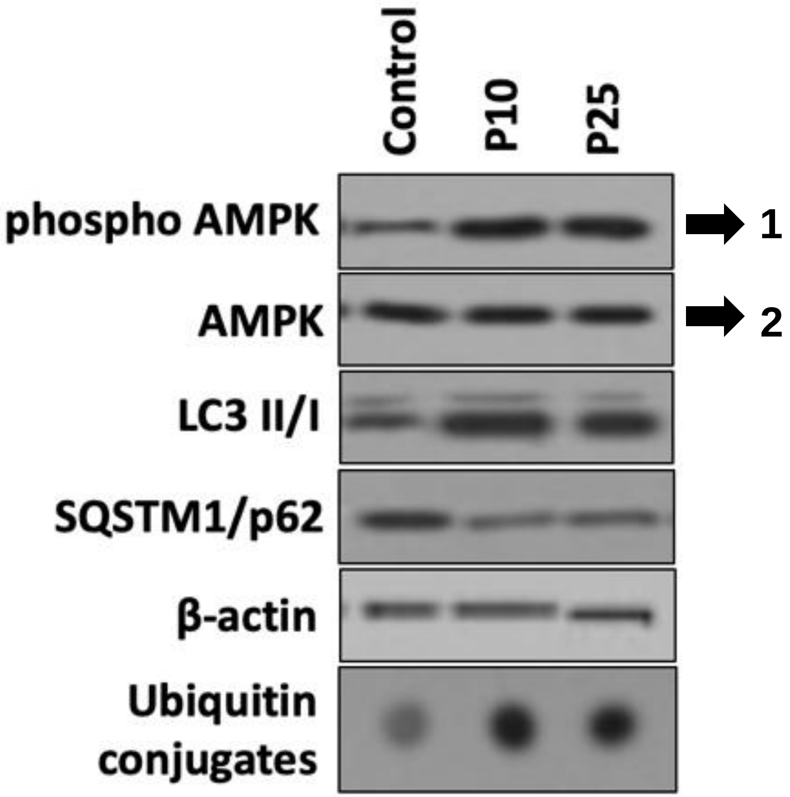

Full unedited immunoblot for figure 4

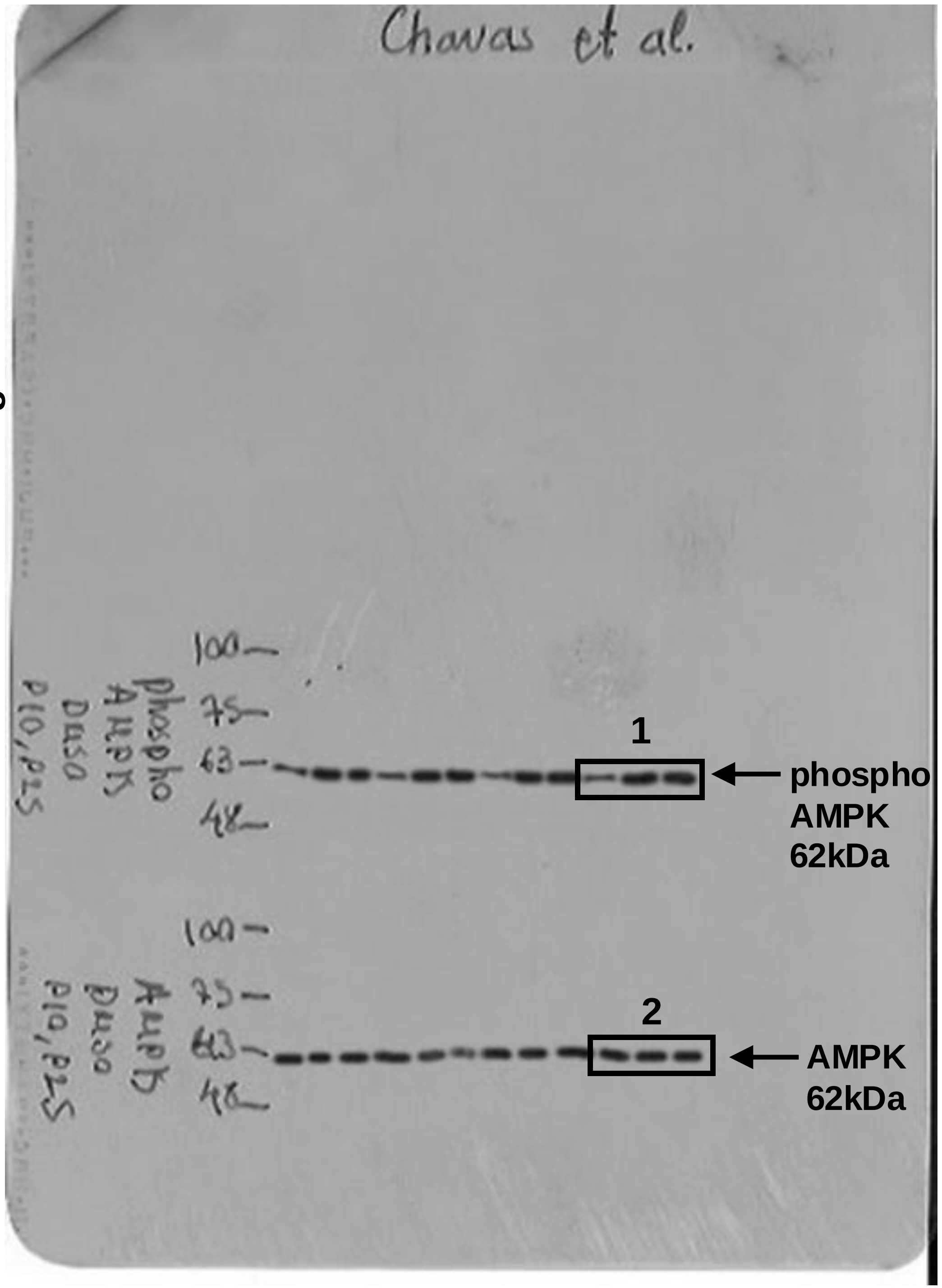

Figure S5: The complete original immunoblots shown in Figure 4 regarding SQSTM1/p62 and LC3 II/I are presented in order below. The individual parts comprising Figure 4 are specified using black boxes.

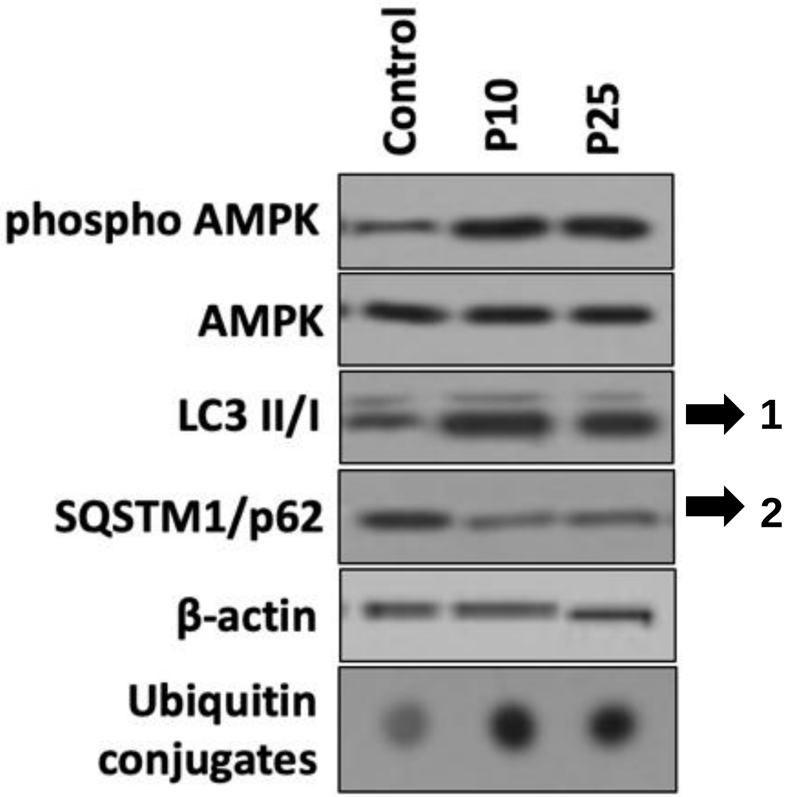

Full unedited immunoblot for figure 4

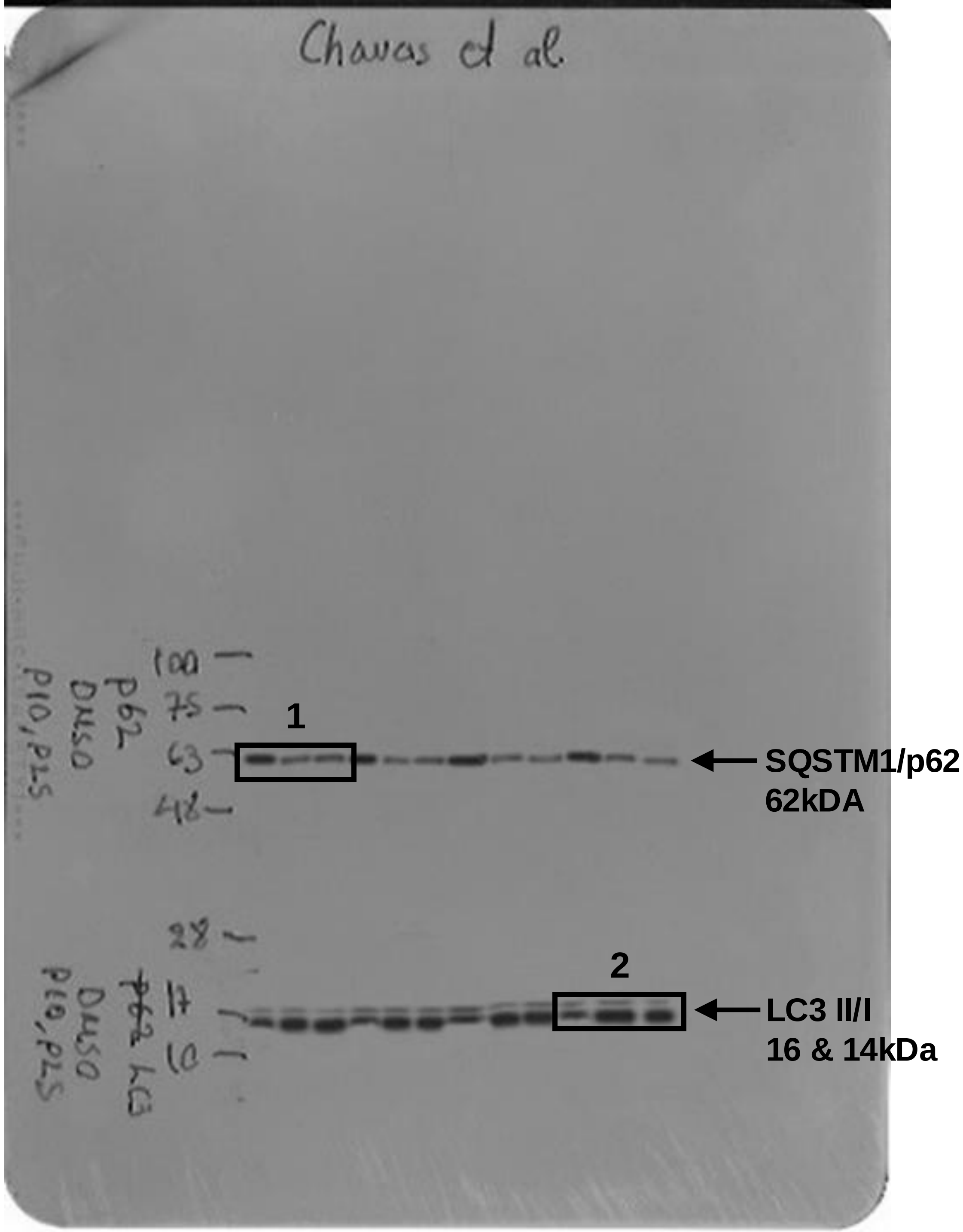

Figure S6: The complete original immunoblots shown in Figure 4 regarding β-actin are presented in order below. The individual parts comprising Figure 4 are specified using black boxes.

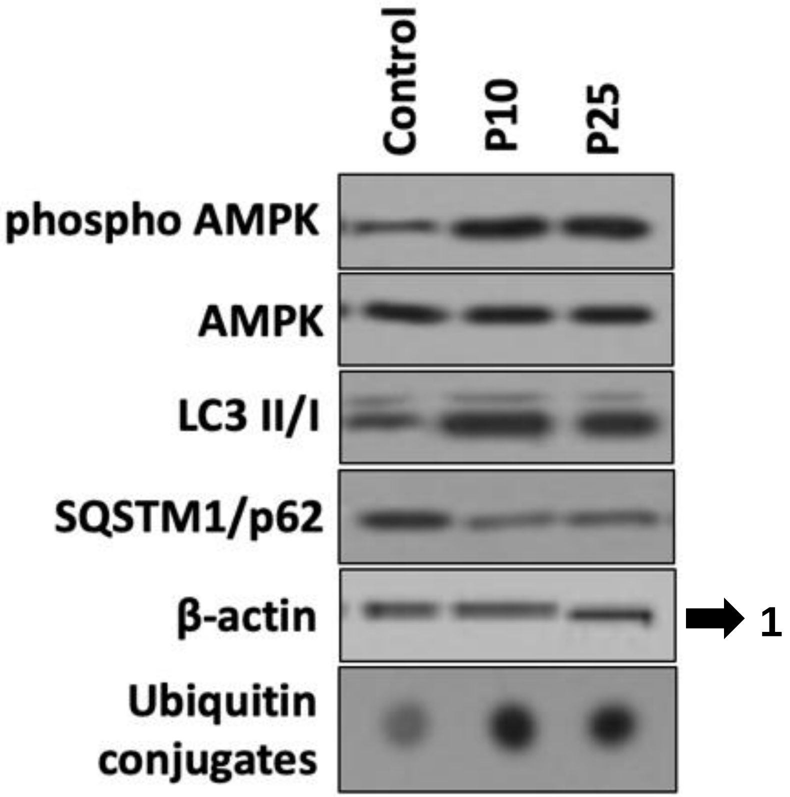

Full unedited immunoblot for figure 4

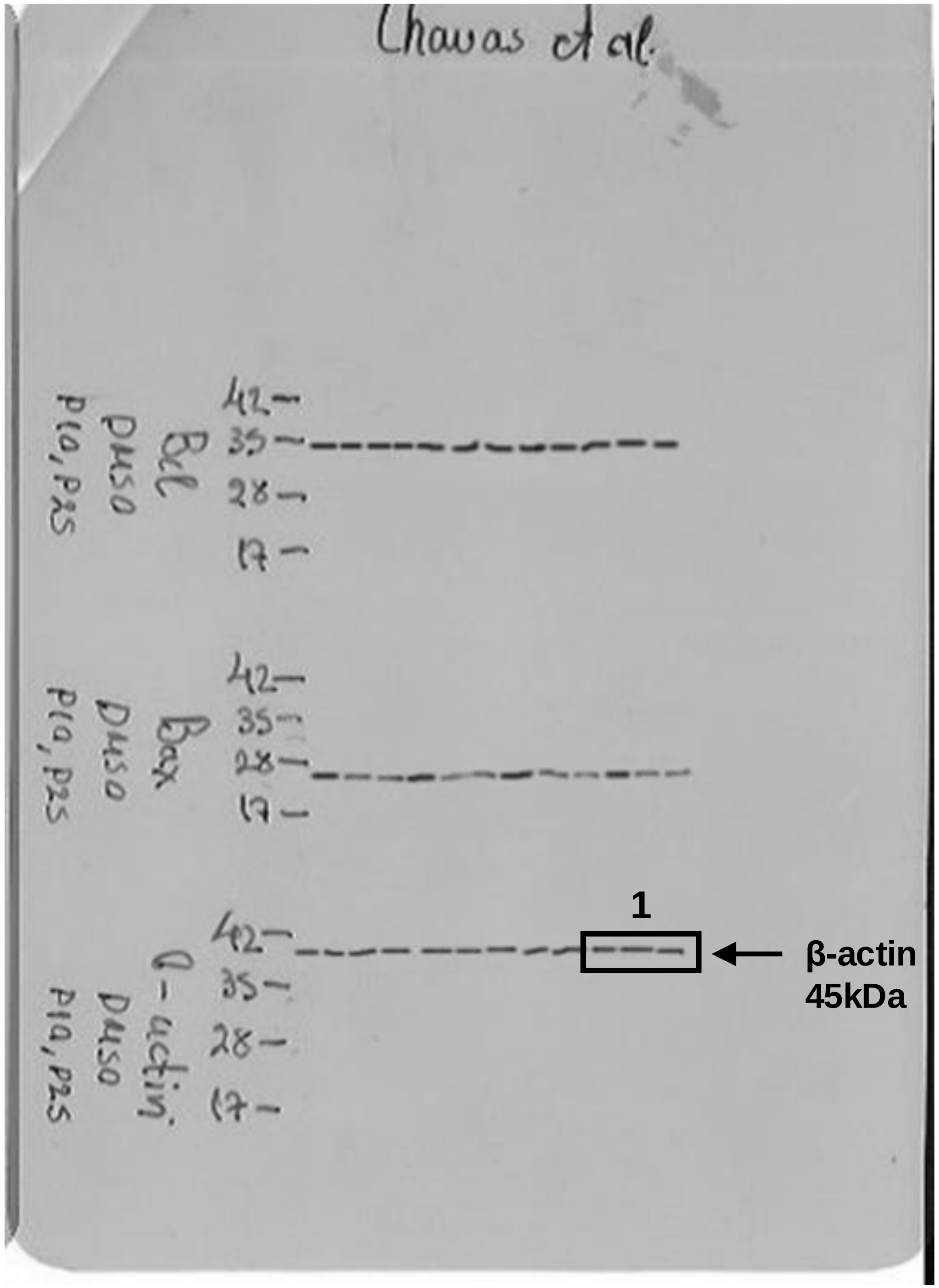

Figure S7: The complete original immunoblots shown in Figure 4 regarding ubiquitin conjugates are presented in order below. The individual parts comprising Figure 4 are specified using black boxes.

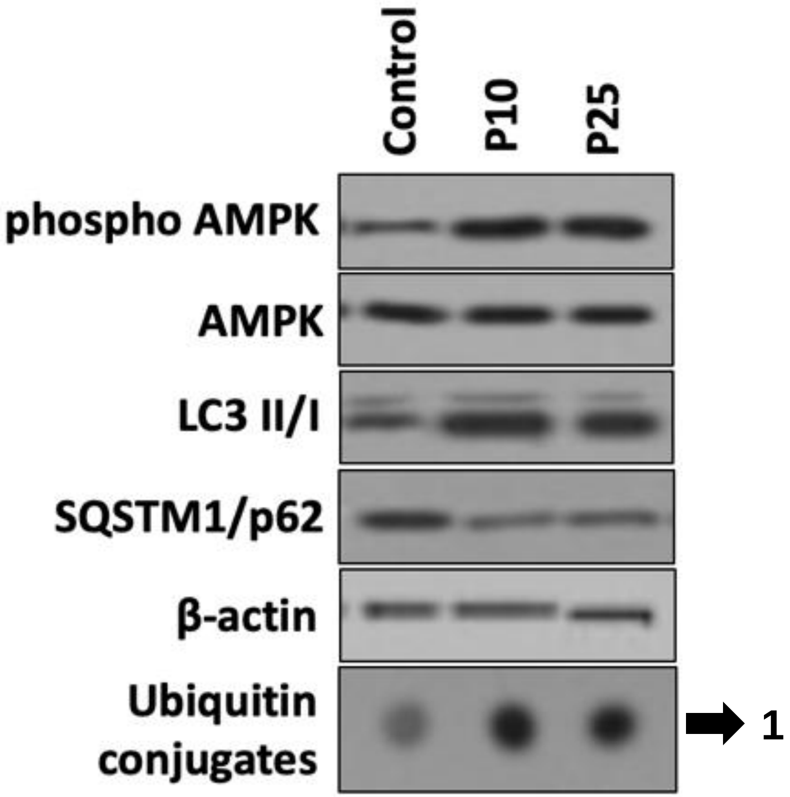

Full unedited immunoblot for figure 4

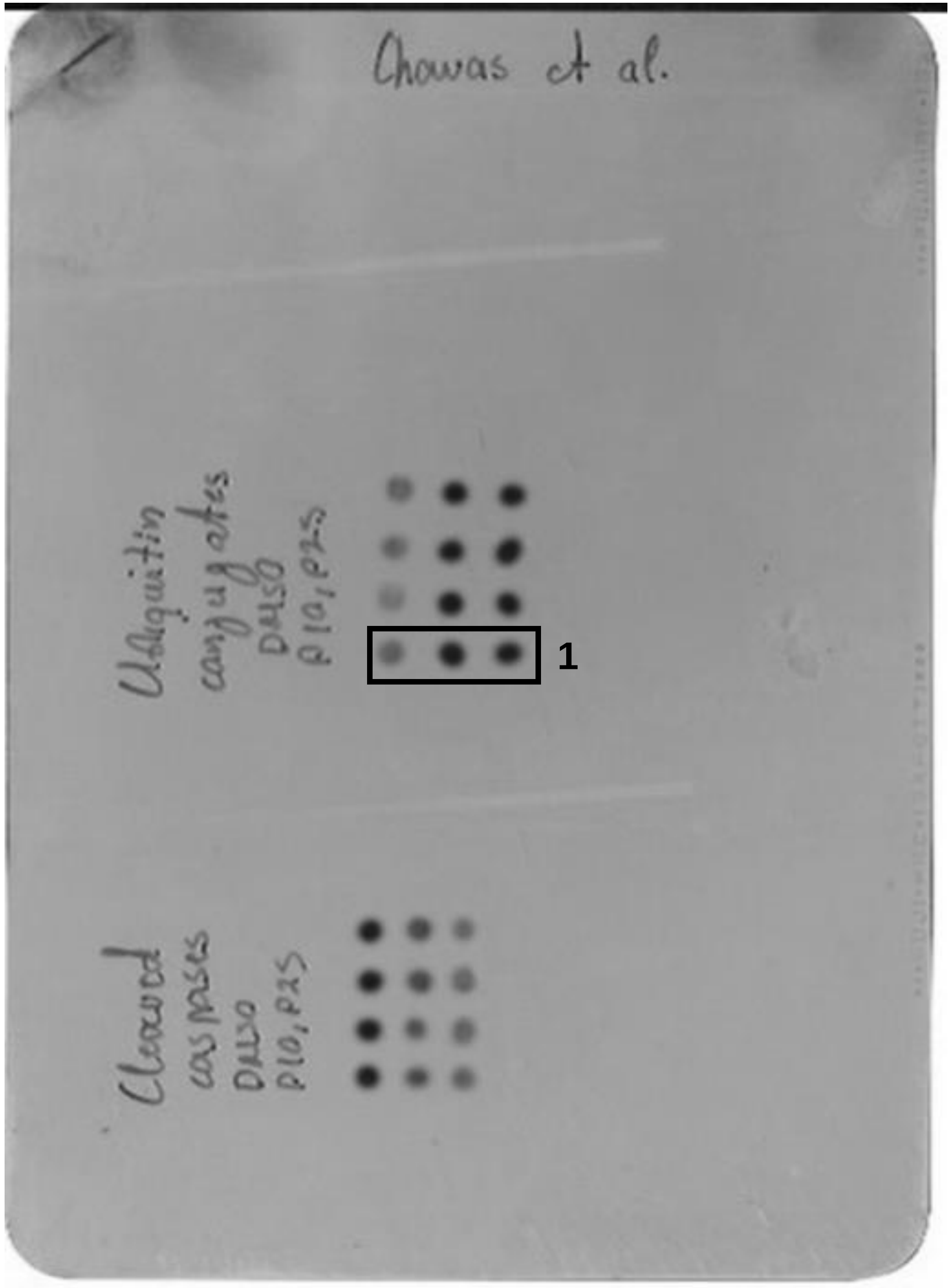

Supplement: Supplementary file 1 [file antioxidants-13-01437-s001.zip › antioxidants-3302694-supplementary.pdf]
